# Effects of COVID-19 on bone fragility: a new perspective from osteoimmunological biomarkers

**DOI:** 10.3389/fimmu.2024.1493643

**Published:** 2024-11-07

**Authors:** Emanuela Galliera, Luca Massaccesi, Laura Mangiavini, Elena De Vecchi, Francesca Villa, Massimiliano Marco Corsi Romanelli, Giuseppe Peretti

**Affiliations:** ^1^ Department of Biomedical Sciences for Health, Università degli Studi di Milano, Milan, Italy; ^2^ IRCCS Istituto Ortopedico Galeazzi, Milan, Italy; ^3^ Laboratorio sperimentale ricerche Biomarcatori Danno d’Organo, IRCCS Istituto Auxologico Italiano, Milan, Italy; ^4^ Department of Experimental and Clinical Pathology, IRCCS Istituto Auxologico Italiano, Milan, Italy

**Keywords:** COVID-19, osteoimmunological markers, bone remodeling, bone fragility, serum biomarkers

## Abstract

**Introduction:**

While there is an increasing understanding of COVID-19's effect on different organs, little is known about the effect of the disease on bone turnover and remodeling so far. Osteoimmunological biomarkers have been described as potential indicators of bone remodeling in inflammatory conditions, but their potential role in evaluating the effect of COVID-19 on bone fragility has not been explored so far.

**Methods:**

The present study aims to measure the osteoimmunological biomarkers in elderly patients undergoing orthopedic surgery, to evaluate the potential effect of COVID-19 on the bone response to the surgery.

**Results:**

In our patients, the RANKL/OPG ratio indicated an increase of bone resorption in COVID-19-positive patients, confirming a strong diagnostic and prognostic value. RANKL/OPG displays a good correlation with the bone fragility maker FGF23, indicating that this parameter is a reliable maker of bone fragility in COVID-19 patients and could provide useful and comprehensive information about inflammation-induced bone loss. Consistently, the RANKL/OPG ratio showed a good correlation also with the two inflammatory markers IL-6 and sRAGE.

**Discussion:**

Taken together these results indicate that the use of an osteoimmunological biomarker like the RANKL/OPG ratio could provide a significant improvement in the clinical evaluation of the COVID-19 effect on bone loss. This aspect is extremely important in elderly patients undergoing orthopedic surgery, which can manifest more severe effects of COVID-19 and present an increased level of age-induced bone fragility.

## 1 Introduction

Coronavirus disease 2019 (COVID-19) is an infectious disease caused by a novel coronavirus (SARS COV-2 or severe acute respiratory syndrome coronavirus) and it was classified as a global pandemic by the World Health Organization (WHO) in March 2020 (1). According to WHO, the number of confirmed cases was over 772.000.000 and the resulting deaths were nearly 7.000.000 (2) as of the end of 2023. From the beginning of the pandemic, an extensive effort was directed to understand the pathogenesis and the clinical effects of COVID-19. Nowadays it is known that COVID-19 includes a wide array of symptoms, but the most common are fever, and fatigue, which can have detrimental pulmonary effects and lung damage. In addition to direct pulmonary damage, COVID-19 has also systemic effects, affecting the cardiovascular system, kidney (3, 4), and the musculoskeletal system with arthralgia and myalgia (2, 5). While there is an increasing understanding of COVID-19’s effect on different organs, little is known about the effect of the disease on bone turnover and remodeling so far. A hallmark of COVID-19 is an acute increase in multiple inflammatory cytokines called “cytokine storm” (6). An increasing number of evidence demonstrated the direct interplay between immune and bone systems, thus leading to a new research field called osteoimmunology. Osteoimmunology is becoming increasingly important for understanding the pathogenesis and developing new therapeutic strategies for, diseases that affect both systems (7). The milestone of osteoimmunology is the RANKL/RANKL/OPG system (8). RANKL/RANK signaling regulates osteoclast formation, activation, and survival in normal bone modeling and remodeling and a variety of pathologic conditions characterized by increased bone turnover. OPG protects bone from excessive resorption by binding to RANKL and preventing it from binding to RANK. Thus, the relative concentration of RANKL and OPG in bone is a major determinant of bone mass and strength (9). Osteoimmunological molecules have been described as potential biomarkers of bone remodeling indicators in inflammatory conditions (10–14), but their potential role in evaluating the effect of COVID-19 on bone fragility has not been explored so far. As COVID-19 has existed for a short time, many effects of the disease still need to be elucidated. Moreover, COVID-19 exerts its major adverse effect on elderly people (15), who are more susceptible to bone fragility. Therefore, the impact of COVID-19 on bone remodeling and bone loss in the elderly is a key issue in the future clinical approach to this disease. In particular, bone fragility is crucial in patients undergoing orthopedic surgery, such as prosthetic arthroplasty, which needs bone remodeling to restore bone tissue homeostasis. To this purpose, the present study aims to measure the osteoimmunological biomarkers in elderly patients undergoing orthopedic surgery, to evaluate the potential effect of COVID-19 on the bone response to the surgery. This could be useful in clinical practice to understand whether the comorbidity of COVID-19 could affect the outcome of orthopedic surgery.

## 2 Materials and methods

### 2.1 Study design and participants

We conducted an observational single-center study from April 2021 to April 2023. Patients admitted to the IRCCS Galeazzi Orthopedic Institute for fracture were included in the study and divided into two groups: patients with COVID-19 infection and patients without COVID-19, both ascertained by nasopharyngeal swab during the patients’ clinical routine.

Inclusion criteria were age greater than or equal to 50 years, patients referred to the emergency room for fracture of the proximal femur, subjects of both sexes, pathologies linked to bone fragility (osteoporosis, fractures, parathyroid pathologies), the signature of informed consent, having performed the nasopharyngeal swab as per clinical practice to verify SARS Cov2 infection. Exclusion criteria were previous conditions of hypovitaminosis D, steroid therapy, presence of autoimmune diseases or those that can create alterations in the inflammatory response.

**Figure 1:**
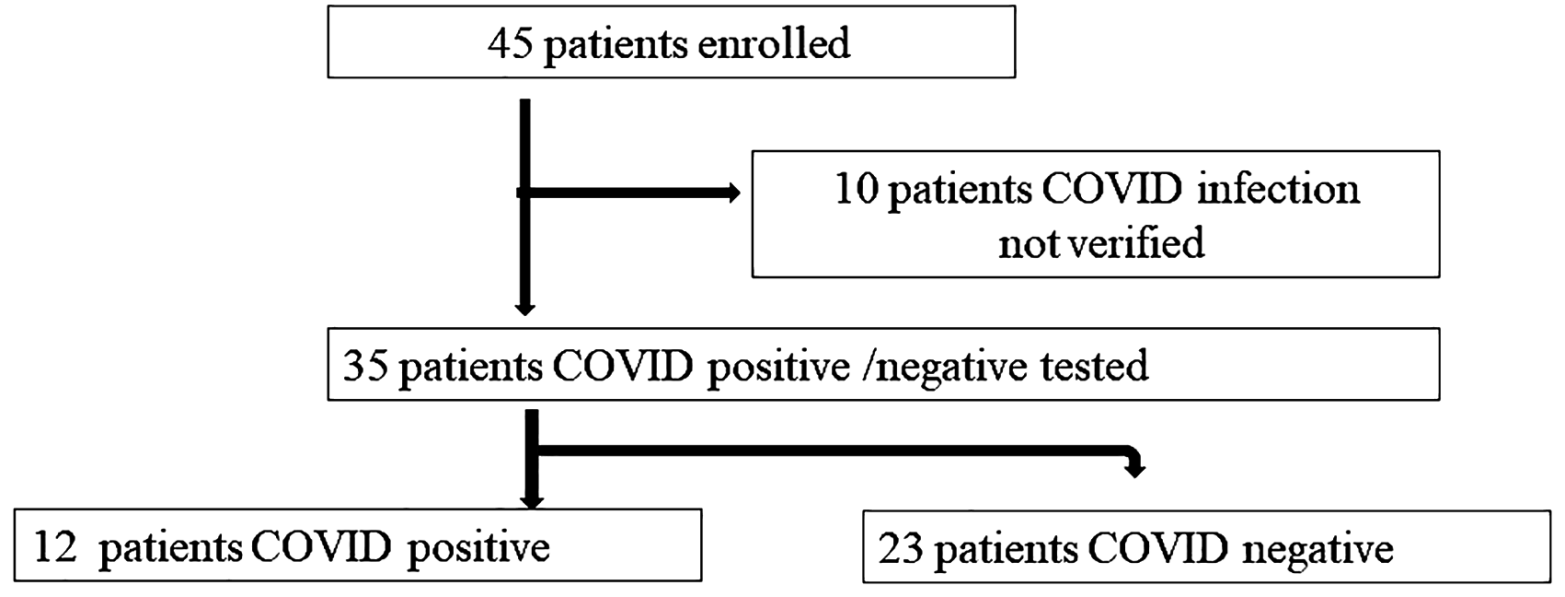
Patients’ enrollment. Patients’ enrollment flow chart and subdivision in two groups, according to the presence or absence of COVID-19 infection.

The flow chart of patient enrollment is described in **Figure 1**: 45 patients (12 male 33 female, age 80,25 ± 10,84) were initially enrolled. Then 10 patients were ruled out for lacking COVID-19 infection positive/negative diagnosis, so the remaining 35 patients (11 Male, 24 female, age 80,51 ± 11,39) were further analyzed in the study. The clinical characteristics of the patients are described in **Table 1**.

**Table 1:** Clinical characteristics of the patient’s population (n=35), divided according to the presence or absence of COVID-19 infection.

Clinical parameters					
	Covid -	Covid +	(t Test) p	Odds Ratio (IC 95%)	significance
	Value ± SD	Value ± SD			
**age**	80.77 ± 11.22	79.02± 9.6	> 0,05		ns
**sex**	7 male 16 female	4 male 8 female		1.095 0.985	ns ns
**Hb (Hemoglobin)**	13,2 g/dL ±1.2	14,1 g/dL ±0.9	> 0,05		ns
**HTC (Hematocrite)**	40,5% ±0.3	39,2% ±0.7	> 0,05		ns
**PLT (Platelet count)**	235000 ±85	211000 ±120	> 0,05		ns
Orthopedic diagnosis					
**Subtrochanteric fracture of the femur**	13	7		1.032	ns
**Subcapital fracture of the femur**	8	4		0.985	ns
**midcervical femoral fracture**	2	1		0.985	ns
Surgical intervention					
**Endoprosthesis**	6	3		0.985	ns
**Intramedullary nail**	13	8		1.022	ns
**Cannulated screws**	1	1		0.985	ns

ns, not statistically significant.

The determinations were performed on blood analysis conducted in the normal diagnostic flow and using the residual material not used for routine diagnostics. Radiographic investigations were performed routinely (pre-operative and immediately post-operative) on all the patients, as well as laboratory tests required by clinical routine, at different time points: T0 (Pre-operative), T1 (24 hours post-operative day), T2 (3 days post-operative day +/- 1 day).

The research related to human use has complied with all the relevant national regulations, and institutional policies and in accordance with the tenets of the Helsinki Declaration. Informed consent was obtained from all participants. The study was approved by the local ethics committee (CE of IRCCS San Raffaele Hospital, Milan, CE 18/INT/2021 Details that might disclose the identity of the subjects under the study were omitted, in accordance with HIPAA. The study was registered as COVID BONE (NCT05352295) to CLINICALTRIALS.GOV.

### 2.2 Blood sample collection and serum preparation

Blood drawing was performed from all the patients and serum and plasma EDTA were obtained at T0 (Pre-operative), T1 (24 hours post-operative day), and T2 (3 days post-operative day +/- 1 day). Serum samples were collected after whole blood collection, clotting, and centrifugation at 400g for 10 minutes at RT without brake. The undiluted serum was aliquoted and stored in polypropylene tubes. Plasma + EDTA and serum samples obtained were stored at -80°C.

### 2.3 Quantification of osteoimmunological markers

RANKL, and OPG, were measured using an ELISA sandwich Quantikine Assay, according to manufacturer protocol (Pikokine ™ ELISA for OPG, quantitative sandwich ELISA for RANKL and FGF23, MyBioSource, San Diego, CA, USA). IL-6 and sRAGE were measured using an ELISA sandwich Quantikine Assay, according to manufacturer protocol (R&D System, Minneapolis, MN, USA). CRP was measured using immunoturbidimetry on an automated biochemical analyzer (Olympus CRP-Latex assay, Central Valley, PA, USA).

### 2.4 Statistical analysis

For all the parameters analyzed, the normality of the distribution of the groups was verified by KS normality. Data are expressed as the mean standard deviation (SD). Longitudinal evaluation of the biomarkers was performed by Mixed Effect Model analysis (NCSS 2024 software), considering p < 0.05 quite significative (*), p < 0.01 very significative (**), p < 0.001 extremely significative (***). Correlation analysis was measured using PRISM 5.0 software, by performing linear regression analysis between the different groups of data and calculating the 95% confidence interval of the regression line. The Pearson correlation coefficient (r2) was calculated to determine the correlation between values measured by the different assays. Statistical analysis of Receiver Operating Characteristic (ROC) curves and Area Under the Curve (AUC) was performed by PRISM 5.0 software.

## 3 Results

### 3.1 Osteoimmunological biomarkers and bone fragility evaluation

**Figure 2:**
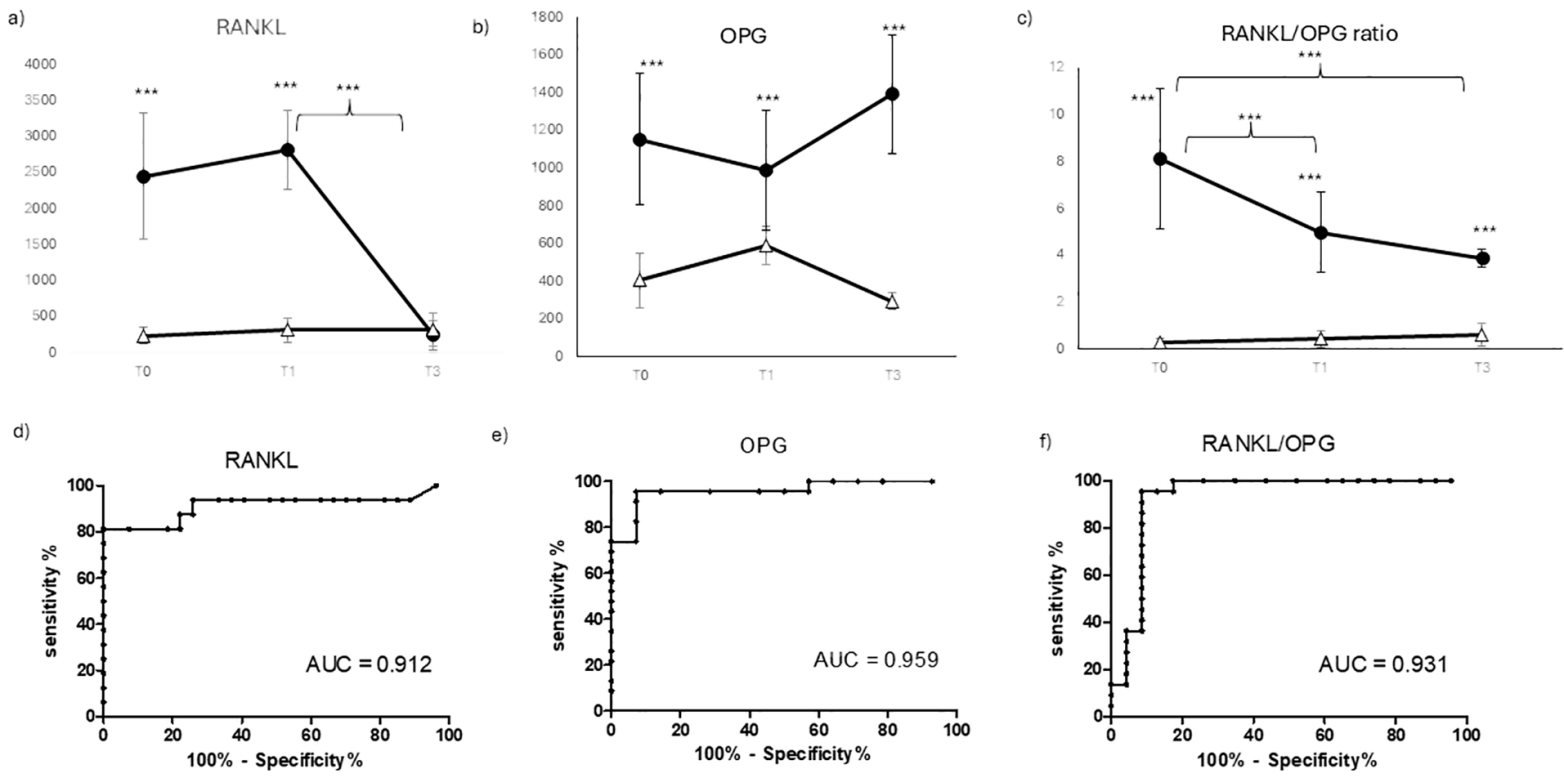
Osteoimmunological biomarkers longitudinal evaluation. Longitudinal evaluation of RANKL **(A)** OPG **(B)** OPG/RANKL ratio **(C)** and the relative ROC (receiving operating curve)) (RANKL **(D)**, OPG **(E)**, RANKL/OPG ratio **(F)**, respectively) in COVID negative patients (white triangle) and COVID positive patients (black dot) (***p < 0.001 extremely significative).

The level of osteoimmunological biomarkers was evaluated in COVID-19-positive and negative patients at different time points, as shown in **Figure 2**. RANKL (panel A), a marker of inflammatory -induced, bone resorption, was significantly higher in COVID-19-positive patients in the pre-surgery (T0) and early post-surgery time point (T1), while it dropped to a comparable level to COVID-19 negative ones at T2. In COVID-19-negative patients, RANKL displays very low levels at any time point. Conversely, OPG (panel B), a bone protective marker, resulted significantly higher in COVID-19 negative patients at any time point, with some not significant fluctuation over time. In COVID-19-positive patients, OPG resulted significantly lower at any time point, with no significant difference over time. The significant difference between the two groups of patients is also confirmed by their ROC AUC, which resulted in very high RANKL (0.912 panel D) and OPG (0.959 panel E). In order to give a comprehensive evaluation, the RANKL/OPG ratio was calculated for the two groups of patients over time (panel C). RANKL/OPG ratio resulted clearly and significantly higher in COVID-19 patients at any time point, with a very high diagnostic value as confirmed by AUC ROC (0.931 panel F). RANKL/OPG also displayed a significative decrease over time, with a weak but quite significant decrease at T1 and a stronger reduction at T1, compared to the pre-surgery time point T0.

**Figure 3:**
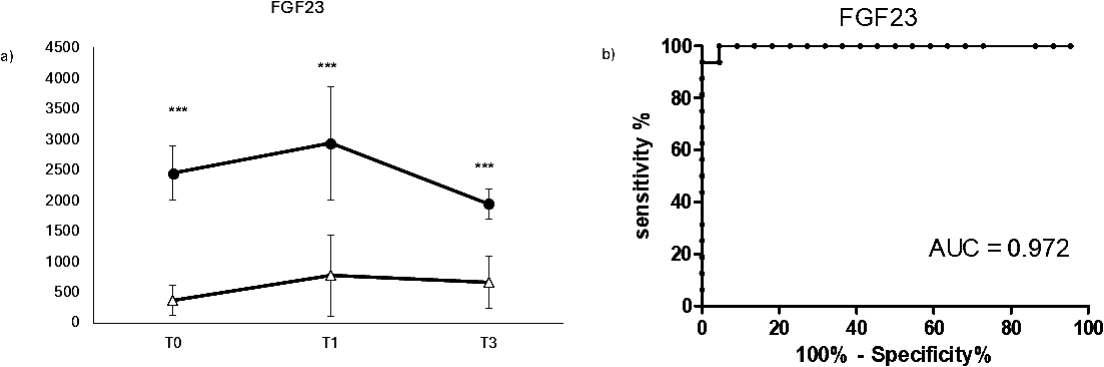
Longitudinal evaluation bone fragility biomarker FGF23. Longitudinal evaluation of FGF23 **(A)** and the relative ROC CURVE (receiving operating curve) **(B)**, in COVID negative patients (white triangle) and COVID positive patients (black dot). (***p < 0.001 extremely significative).

In order to evaluate the impact of COVID-19 on bone metabolism and fragility, the serum biomarker FGF23 was evaluated Covid19 in positive and negative patients at different time points, as shown in **Figure 3**. FGF23 showed a very significant increase in COVID-19-positive patients at any time point (panel A), indicating a good diagnostic potential as confirmed by its ROC AUC (0.972 panel B). On the other hand, FGF23 didn’t show a significant variation over time, suggesting that this molecule could be more suitable in the diagnosis rather than in the early prognosis of Covid19 - induced bone fragility.

### 3.2 Inflammatory markers evaluation

**Figure 4:**
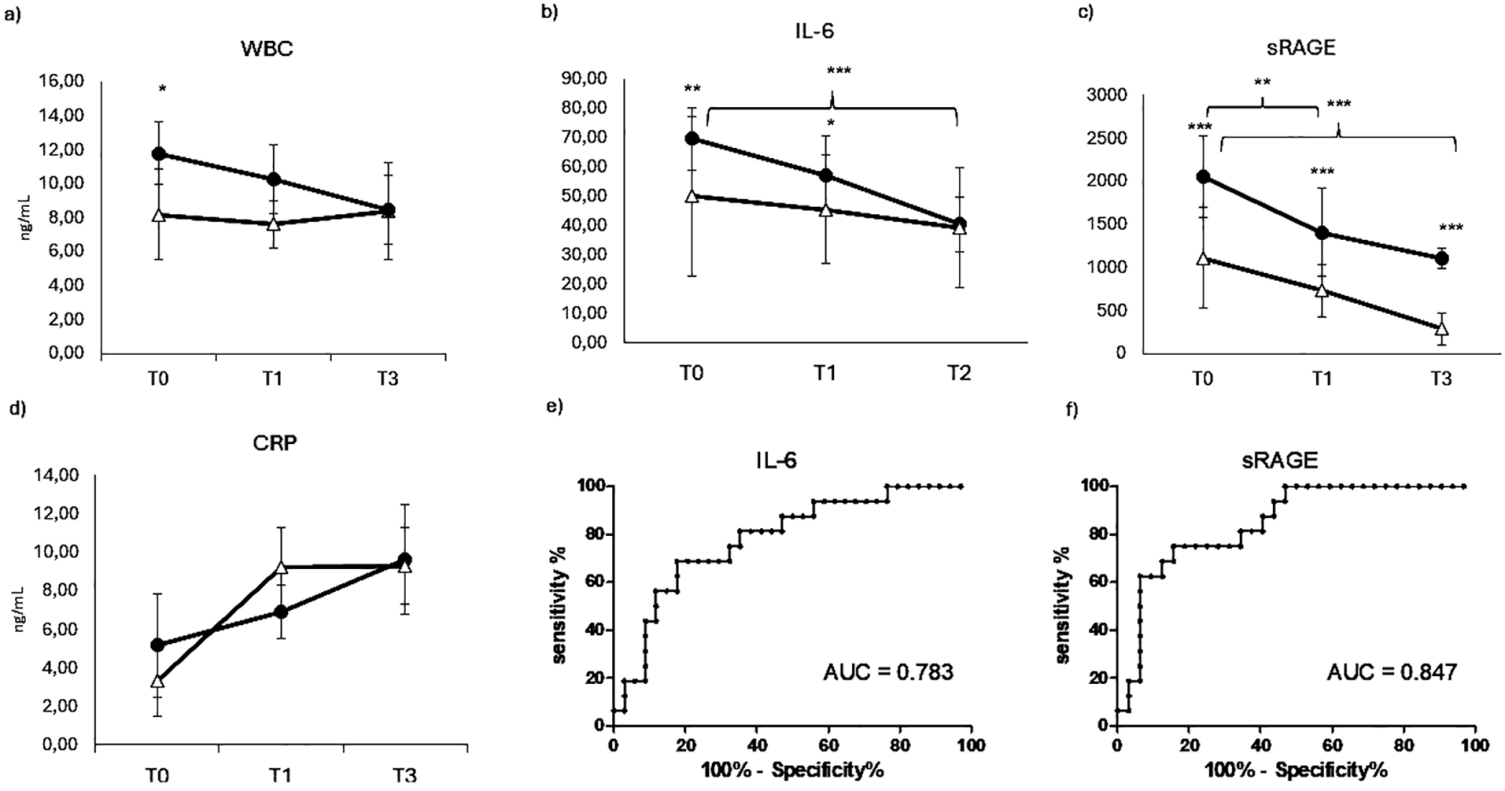
Inflammatory markers evaluation. Longitudinal evaluation of WBC **(A)** IL-6 **(B)** sRAGE **(C)** and CRP (RANKL **(D)**, in COVID negative patients (white triangle) and COVID positive patients (black dot)). (*p < 0.05 quite significative, **p < 0.01 very significative, ***p < 0.001 extremely significative). ROC CURVES (receiving operating curve) of IL-6 **(E)** and sRAGE **(F)**.

In order to evaluate the inflammatory status of the patients, which could affect bone remodeling, routine parameters of inflammation such as White blood cell WBC (**Figure 4A**) and C-reactive protein (**Figure 4D**) were measured in COVID-19 positive and negative patients. Surprisingly, WBC displayed a weak but quite significantly higher level in COVID-19-negative patients compared to positive ones at T0. This difference slightly reduced over time, becoming not significant at T1 and reaching comparable levels between the two groups at T2. Similarly, CRP (**Figure 4D**) displayed higher but not significant level in COVID-19-positive patients compared to negative at T0, while in the early preoperative time point, T1 showed a weak but opposite trend, until reaching a comparable level between the two groups at T2. In any case, there were no significant differences neither between the two groups of patients nor in the longitudinal evaluation. For this reason, the AUC ROC for WBC and CRP were non-significant and were not presented among the results.

Since the canonical inflammatory markers were not informative in the differentiation of our two groups of patients, we evaluated a marker that could provide information about the cytokine response. Among primary cytokines, IL-6 is the main responsible of the systemic effect of inflammation and therefore a very suitable serum maker of inflammatory status. Therefore IL-6 was evaluated in COVID-19 positive and negative patients at different time points, as shown in **Figure 4**. Differently from the previous inflammatory markers, IL-6 displays a significative difference between the two groups of patients, showing a very significative higher level in COVID-19-positive patients at T0. This difference is maintained, even though less significative, at T0, while at the last time point, IL-6 decreases reaching comparable levels measured in COVID-19 patients. IL-6 displayed also a longitudinal value, showing a significative decrease in COVID-19-positive patients over time. This value is confirmed by the IL-6 ROC AUC resulting in a quite good value of 0.783 (panel E).

Blood levels of the soluble receptor for advanced glycation end-products (sRAGE) are acutely elevated during the host inflammatory response to infection and have an important prognostic role in COVID-19. In this study, the diagnostic and prognostic value of sRAGE was evaluated in orthopedic COVID-19-positive and negative patients in order to complete the panel of inflammatory markers. The marker sRAGE displayed a very strong increase in COVID-19-positive patients compared to negative ones at any time point (**Figure 4C**), confirming a very good diagnostic value, as confirmed by a very strong ROC AUC (0.847, panel F). It also displays a very good prognostic value in the longitudinal evaluation, showing a progressive and significant decrease in COVID-19-positive patients over time.

### 3.3 Correlation of osteoimmunological markers with inflammatory and bone fragility makers

**Figure 5:**
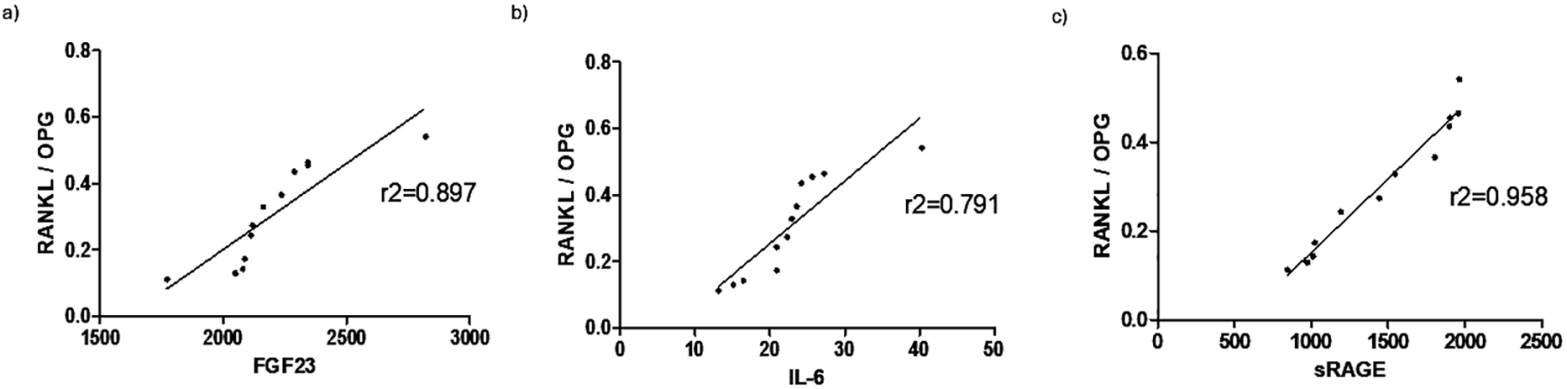
Correlation of osteoimmunological markers with inflammatory and bone fragility makers. Correlation (Spearman r, 95% confidence interval) of RANKL/OPG with the bone fragility biomarker FGF23 **(A)**, and inflammatory biomarkers IL-6 **(B)** and sRAGE **(C)**.

In order to highlight how osteoimmunological biomarkers can describe the link between COVID -19 infection, inflammation and bone fragility, a correlation analysis was performed between the osteoimmunological biomarker RANKL/OPG and the inflammatory marker IL-6 as well as well as the bone fragility marker FGF23, as shown in **Figure 5**. RANKL/OPG displayed a very good correlation with all the three parameters evaluated, resulting in r2 = 0.897, r2 = 0.791, r2 = 0.958 with FGF23 (panel A), IL-6 (panel B), and sRAGE (panel C), respectively.

## 4 Discussion

The role of the virus in bone loss has long been underestimated, but investigation in recent decades revealed the involvement of the virus in bone turnover. The first studies were performed on human immunodeficiency virus (HIV) indicating that infected patients showed an increased prevalence of bone-related disease (16, 17), due to a disruption of the bone-skeleton interface resulting in increased bone fragility and fractures. In the context of SARS-COV infections, the first evidence came from studies on 2003 SARS–COV–1 outbreak: during the previous epidemic of SARS, bone necrosis and decreased bone mineral density (BMD) were reported in infected patients (18). On the basis of this experience, few recent evidence has begun to explore the possible effect of the recent SARS-COV 2 pandemic on bone loss. Few recent studies on murine models of SARS-CoV 2 infection described a significant increase in osteoclast production, leading to bone loss (19, 20). Observation from the pandemic indicates that bone loss and mineral metabolism may be altered with SARS-CoV-2 infection. In particular, initial studies indicate that COVID-19 patients can manifest alteration in mineral metabolism, bone turnover makers, and risk of vertebral fracture (21), but further studies are needed to understand better the mechanism of COVID-19 -induced bone loss, to define the clinical approach to evaluate and monitor this effect. These effects are prominent in elderly patients, therefore the risk of bone loss in these patients should be closely monitored. It is well-recognized that the degree of local and systemic inflammation is related to the degree of bone loss. Therefore, a comprehensive approach to the effect of COVID-19 infection on bone loss could come from osteoimmunology, a new interdisciplinary field that emerged in the last two decades and describes the direct interaction between the skeletal system and host immune system (7). Indeed, bone cells interact with immune cells under physiological and pathological conditions. Osteoimmunology was created as a new interdisciplinary field in large part to highlight the shared molecules and reciprocal interactions between the two systems in both health and disease. The concept of a unified osteoimmune system has become absolutely indispensable for basic and translational approaches to diseases related to bone and/or the immune system. Osteoimmunology has therefore become indispensable for understanding the pathogenesis of a number of diseases, such as rheumatoid arthritis (RA) (9), where the inflammation induces bone erosion and could provide new insight in understanding the effect of host response to COVID-19 on bone.

The hallmark of osteoimmunology is the RANKL/RANK/OPG system, known for its roles in osteoclast maturation, bone modeling, and bone remodeling. The receptor activator of NF-kB (RANK), receptor activator of NF-kB ligand (RANKL), and osteoprotegerin (OPG) are the main components of this signaling system. In the RANKL/RANK/OPG pathway, RANKL binds to RANK as its receptor and eventually leads to osteoclast precursor maturation. Osteoprotegerin is known as a decoy receptor for RANKL which prevents RANKL-RANK binding (22, 23). RANKL and OPG exist as soluble circulating molecules and can be measured as circulating biomarkers of bone remodeling in inflammatory conditions. In our study, RANKL and OPG displayed different behaviors in COVID-19-positive and negative patients. In Covid-positive patients, RANKL, the marker of bone resorption was clearly higher at early time points and, consistently OPG, the bone protective marker, was significantly lower. Only RANKL showed a significative decrease at the last time point, while OPG showed no significant variation over time, indicating that these two markers have a great diagnostic value, as confirmed by their strong ROC AUC, but not a great prognostic value if considered singularly. To overcome this limit, the information of these two markers was combined together with the RANKL/OPG ratio. The RANKL/OPG ratio is critical for guiding bone resorption, and it is considered the ultimate parameter that provides clear information on bone turnover balance in inflammatory conditions. The altered RANKL/OPG ratio has been described in multiple autoimmune diseases and has been linked to decreased BMD (24). In our patients, the RANKL/OPG ratio clearly indicated the increase of bone resorption in COVID-19-positive patients, confirming a strong diagnostic value, as indicated by the strong ROC AUC, and it also added a prognostic value, indicating a slight but significative reduction of bone resorption over time after surgery. The increased bone loss in COVID-19 patients has recently been described in terms of bone mineral density (BMD) evaluation (2). BMD is a good biomarker of bone fragility but is not a practical measurement to take from COVID-19-positive patients during illness (25), therefore a serum biomarker of bone fragility could be easier to measure and less invasive on the patients. Recent studies described the relationship between the serum fibroblast growth factor 23 (FGF23) level and bone fragility (26). In our patients, FGF23 displays a strong increase in COVID-19-positive patients compared to negative ones, with no significative variation over time, suggesting a good diagnostic potential, as confirmed by a very strong AUC ROC. These results also confirmed that the surgery has no impact on the bone fragility status of the patients, which is mainly related to the presence or absence of COVID-19 infection, reinforcing the concept of infection-induced bone loss.

It is interesting to notice that aging is recently regarded as a chronic systemic inflammatory condition, named “inflammaging”, and characterized by metabolic dysregulation and the increase of several inflammatory biomarkers (27, 28). Mechanisms involved in the pathogenesis of “inflammaging” are multiple, but the crucial input is the continuous stimulus of internal activation in “inflammaging” that makes immune cells less responsive and predisposes patients with “inflammaging” to infectious complications, such as COVID-19 (29). Thus, a derangement in bone niche function interfering with the intense crosstalk between immune cells and bone cells is likely. Our patients are mainly elderly people undergoing major orthopedic surgery, therefore the potential effect of COVID-19 on their bone status is important to the clinical outcome. To this purpose, the inflammatory status of COVID-19 positive and negative patients was evaluated, but surprisingly the canonical biomarkers in inflammation, such as WBC and C- C-reactive protein (CRP), display little if no difference between the two populations, with a slightly but not significative increase in COVID 19negative patients. This result could appear contradictory, but since the overall level of CRP and WBC are very low, the results of these biomarkers can be more due to a general inflammatory response to surgery rather than a host response to the infection. Recent findings of our group showed that CRP is not a preferential marker of COVID-19 (30): this study was conducted on ICU patients, therefore this consideration is even more applicable to less severe conditions of infection, as in the case of COVID -19 patients of the present study. These results suggest the need for different makers of inflammation that could be more informative. A significant role in the COVID-19 cytokine storm is played by the inflammatory cytokine IL-6 (31), acting as a major player in the systemic effect of pro-inflammatory acute inflammatory response. Moreover, IL-6 has been recently described as a COVID-19 severity predictor (32–35). Therefore, IL-6 was evaluated in this study in COVID-19-positive and negative patients over time. IL-6 confirmed a good diagnostic power, showing quite a significant increase in COVID -19 Patients at any timepoint. IL-6 also confirmed a significant prognostic value, showing a significant decrease in COVID-19 patients at later time points. The increase of IL-6 in COVID-19-positive patients compared to negative ones is not as striking as in patients requiring ICU (30) confirming that the inflammatory response to SARS-COV 2 infection is not as extreme as in severe conditions. All the patients received at least two doses of anti-COVID-19 vaccine at the moment of the enrollment, so this could explain the absence of extremely severe inflammation in response to the infection, but still able to affect bone remodeling.

There are multiple mechanisms for how COVID-19-induced inflammation can affect bone status. Besides osteoimmunological biomarkers production, inflammation can result in an increase of reactive oxidative species (ROS) which may cause protein damage and an increase of advanced glycation end-products (AGEs) (36). AGEs can have detrimental effects on crosslinks in bone, destabilizing bone structure (37). Therefore, inflammation, occurring in COVID-19, induces changes in the bone microenvironment thus having a direct effect on bone composition and strength. Furthermore, it has been observed that during inflammation, the activation of receptors of AGEs (RAGE) can increase osteoclastogenesis (38, 39). In particular, the soluble receptor of AGEs (sRAGE) has been recently described as a biomarker of COVID-19 disease severity (40). Being present as a soluble form in circulation, sRAGE can be easily measured and has already been described as a biomarker in several diseases, ranging from cardiovascular to renal and liver disorders and sepsis (41). In our patients, sRAGE confirmed a good diagnostic and prognostic value in COVID -19 positive patients, showing a good AUC ROC curve and a significative reduction in sRAGE from a high level over time. Oxidative stress has been found to induce apoptosis of osteocytes and osteoblast through the regulation of RANKL and OPG, inducing osteoclastogenesis and leading to bone loss (42, 43). In agreement with these reports, in our patients, sRAGE displays the same behavior of RANKL/OPG ratio, with a significant increase in COVID-19-positive patients and a progressive decrease over time.

In order to show the potential value of osteoimmunological biomarkers to provide a direct hint about the correlation between COVID-19 infection, inflammation, and the resulting bone remodeling, a correlation analysis was performed between RANKL/OPG, and the most significative biomarkers evaluated, as shown in **Figure 5**. RANKL/OPG provides direct information about the balance between bone formation and resorption and displays a good correlation with the bone fragility maker FGF23, indicating that this parameter is a reliable maker of bone fragility in COVID19 patients and could provide useful and comprehensive information about the inflammation-induce bone loss. Consistently, the RANKL/OPG ratio showed a good correlation also with the two inflammatory parameters that resulted significantly in this study to highlight the inflammatory response to COVID-19, namely IL-6 and sRAGE: IL- 6 as a systemic primary inflammatory cytokine involved in the cytokine storm resulting in COVID 19 disease, sRAGE as a predictive biomarker of COVID -19 severity. RANKL/OPG showed a good positive correlation with IL-6 and a very strong positive correlation with sRAGE. This is consistent with the trend over time of both RANKL/OPG and sRAGE, which displayed not only a significative difference between COVID-19 positive and negative patients but a progressive decrease over time, therefore suggesting both a diagnostic and prognostic value. These results indicate that RANKL/OPG can reflect not only the bone status but also the inflammatory status of the patient, confirming the intrinsic capacity of osteoimmunology to link bone and the immune system.

The limits of the study can be represented by the number of patients and the time points examined. The limits of COVID-19 patients are due to the difficulty of recruiting COVID-19 patients undergoing orthopedic surgery. The kind of surgery considered was not an emergency one so it could be delayed for a long time in case of infection. Moreover, at the time of recruitment, there was more awareness about the COVID-19 protocols and preventive measures, therefore a patient who accidentally discovered to be positive would just delay the surgery. There the positive patients enrolled in the study were those found incidentally positive in the routine pre-surgery nasopharyngeal swab test. As far as the time points are concerned, being an observational study, they were evaluated according to the orthopedic surgery clinical protocol, but the author cannot exclude that longer time points would provide a more significant longitudinal value for the biomarkers evaluated over time.

Even considering this limitation, the results of this study clearly indicate that the use of an osteoimmunological biomarker like the RANKL/OPG ratio could provide a significant improvement in the clinical evaluation of the COVID-19 effect on bone loss. This aspect can be extremely important in those elderly patients undergoing orthopedic surgery, which, on the one hand, can manifest, more severe effects of COVID-19 and, on the other hand, present an increased level of age-induced bone fragility.

The evolution of COVID-19 to an endemic state would mean that COVID-19 continues to circulate in the population at a steady rate. This could happen through a combination of factors such as widespread immunity from vaccination or previous infection, along with the virus becoming less severe over time, but specific attention should be addressed to the elderly. Indeed, COVID-19 had a particularly significant impact on the elderly population. Older adults have been identified as being at higher risk of severe illness and death from COVID-19 compared to younger age groups. The elderly population is also the one undergoing bone surgery more frequently and, as the global population continues to age and life expectancy increases, the demand for orthopedic surgeries among the elderly is expected to rise. Elderly individuals often have pre-existing conditions that can make them more susceptible to complications from COVID-19. Many older adults with long COVID experience persistent inflammation, fatigue, and musculoskeletal pain. This ongoing inflammation can worsen inflammaging, maintaining a cycle of bone loss and increasing the risk of fractures, particularly in already fragile elderly individuals Age-related changes in bone density and immune function can exacerbate the impact of the virus. Moreover, prolonged inflammation and immune dysregulation associated with severe COVID-19 might accelerate bone loss or exacerbate conditions like osteoporosis. In addition, elderly people with COVID-19 often experience long periods of immobility due to hospitalization or fatigue during recovery. Lack of physical activity can lead to muscle weakness and bone demineralization, increasing the risk of falls and fractures. Recovery from severe COVID-19 may lead to post-acute sequelae (long COVID), including persistent fatigue, which further reduces mobility and bone strength over time. The combination of decreased bone density, reduced mobility, and general frailty makes elderly COVID-19 survivors more prone to fractures. Hip and vertebral fractures are particularly common and can have devastating impacts on quality of life and survival rates in this population. One of the aims of this study is to understand better how COVID-19 affects bone health and to identify ways to mitigate these effects. To this purpose, osteoimmunology offers valuable perspectives on how COVID-19 might affect bone health, especially in the elderly. The interplay between immune responses and bone metabolism highlights the importance of managing inflammation and maintaining bone health in vulnerable populations during and after the pandemic. In order to mitigate the bone fractures, vitamin D and calcium supplementation, could support bone health, as well as regular check of bone fragility in elderly individuals post-COVID, particularly those with long-term symptoms or steroid treatments. Addressing these issues is essential in supporting the elderly during recovery from COVID-19, given their heightened vulnerability to bone-related complications. The use of an osteoimmunological biomarker that could provide information about both the inflammatory and bone fragility status at the same time would have important implications in the clinical management of COVID-19-related complications affecting bone health. Potential interventions could include targeted treatments to manage inflammation and promote bone health during and after COVID-19 infection.

## Data Availability

The original contributions presented in the study are included in the article/supplementary materials, further inquiries can be directed to the corresponding author/s.
